# Bridging personality, time poverty, and rest intolerance: a network analysis

**DOI:** 10.3389/fpsyg.2026.1872834

**Published:** 2026-07-17

**Authors:** Haoyu Wang, Ling Cheng, Wenyan Cheng, Bohan Wang

**Affiliations:** 1 College of Education Science, Anqing Normal University, Anqing, Anhui, China; 2 Jing Hengyi School of Education, Hangzhou Normal University, Hangzhou, Zhejiang, China

**Keywords:** big five personality, network analysis, perceived time poverty, rest intolerance, state anxiety

## Abstract

**Introduction:**

Rest intolerance—the inability to rest without experiencing negative emotions or cognitive distress—is an emerging psychological phenomenon. This study aimed to explore the complex interplay among personality traits, state anxiety, perceived time poverty, and rest intolerance.

**Methods:**

Using a sample of 541 college students, we constructed a 13-node regularized partial correlation network at the dimension level, excluding one unreliable subscale.

**Results:**

Results revealed that Neuroticism, Time Pressure, and Negative Feelings emerged as the central nodes within their respective psychological domains. Crucially, bridge centrality analysis identified State Anxiety, Neuroticism, Negative Feelings, and Time Pressure as the core bridge nodes connecting the broader psychological network.

**Discussion:**

These findings clarify a “trait-cognition-emotion” interactive pattern where individuals with high neuroticism are particularly susceptible to internalizing time pressure and state anxiety, which is subsequently associated with rest intolerance. This study elucidates the micro-level structural relationships of rest intolerance and provides plausible targets for future intervention studies to mitigate rest intolerance among vulnerable individuals.

## 1 Introduction

In today's fast-paced society, which valorizes the “Entrepreneurial Ethic” (Harrell, 1985), the meaning of rest is profoundly alienated. Although high-quality leisure is crucial for physical and mental recovery, well-being, and cognitive function (Sonnentag and Zijlstra, 2006; Sonnentag and Fritz, 2007; Kuykendall et al., 2015), many individuals are “unable to rest with peace of mind”. This phenomenon is particularly pronounced among college students facing fierce academic competition (Koo, 2023). Beyond objective burdens, they experience intense guilt, shame, or anxiety during rest, often compulsively thinking about unfinished tasks (Liu et al., 2023). Recently, Wang et al. (2025) formally conceptualized these negative experiences and cognitive biases as Rest Intolerance. They identified four dimensions of rest intolerance within the Chinese context: Negative Feelings, Obsessive Thinking, Social Comparison, and Cognitive Bias. It is crucial to distinguish rest intolerance from related constructs. While leisure guilt primarily emphasizes moral self-blame regarding time use, and productivity anxiety focuses on apprehension about future tasks, rest intolerance constitutes a distinct, immediate affective-cognitive symptom cluster triggered specifically by the physical cessation of work (Wang et al., 2025). It captures not just the guilt, but the profound inability to psychologically detach and tolerate the resting state itself.

Theoretically, the inability to rest can be understood as a severe impairment in the psychological recovery process. Classic occupational and educational psychology literature extensively emphasizes the necessity of psychological detachment—the ability to mentally disengage from work or study-related thoughts during off-job time (Sonnentag and Zijlstra, 2006; Sonnentag and Fritz, 2007). A failure to detach not only prevents the restoration of depleted cognitive and emotional resources but also significantly contributes to academic burnout and chronic stress among students (Walburg, 2014; May et al., 2018). Building upon this tradition, recent conceptualizations argue that this impairment extends beyond mere cognitive rumination. The phenomenon of 'leisure guilt' further captures the moral and emotional distress individuals experience when engaging in non-productive activities, highlighting a profound sociocultural alienation of rest (Tonietto et al., 2021; Koo, 2023). Thus, rest intolerance represents the extreme manifestation of this broader crisis in psychological recovery, encapsulating not only the failure to detach but also the immediate affective resistance to rest itself. Despite growing attention, systematic empirical inquiry into its associated factors remains scarce, particularly its interactions with personality traits, emotional states (e.g., anxiety), and time cognition (e.g., perceived time poverty).

Perceived Time Poverty is a significant cognitive correlate for rest intolerance. Unlike objective time scarcity, it is a subjective negative experience arising from heavy task loads, encompassing Time Pressure, Time Urgency, and Academic & Leisure Conflict (Cheng et al., 2025). Time poverty significantly associated with negative emotions such as depression and anxiety (Bond and Feather, 1988; Macan et al., 1990). According to the Conservation of Resources Theory (Hobfoll, 1989), individuals perceiving severe time scarcity view non-productive rest as a waste. Koo (2023) further indicates that this excessive focus on time productivity leads to “Leisure Guilt,” undermining leisure enjoyment and turning rest into a psychological burden. For college students, feeling time-poor is closely linked to cognitive resistance to rest; the more time-poor they feel, the less they can tolerate stopping.

However, perceived time poverty frequently intertwines with State Anxiety. As a high-arousal negative emotion, anxiety alters perceptual judgments of physical environments and personal resources, magnifying perceived risks and losses (Blanchette and and Richards, 2010). Chen et al. (2023) found that anxiety depletes psychological resources, biasing perceptions of environmental threats. Applied to the domain of rest, high state anxiety distorts assessments of relaxation, framing it as a threatening sign of “falling behind” or “losing control”. This exacerbates obsessive thinking and social comparison during rest, which are characteristic of rest intolerance (Li, 2002).

At a deeper level, this susceptibility may root in Personality Traits. From a transdiagnostic perspective (Widiger and Oltmanns, 2017) and Eysenck's biopsychological model, Neuroticism acts as a fundamental, distal vulnerability factor that predisposes individuals to chronic physiological hyperarousal and negative emotionality. Yang et al. (2025) suggest that personality traits interact with emotional states through specific “bridges” rather than single pathways. Rather than directly determining specific disorders, such broad traits provide a pathological background. Thus, personality traits likely act as distal background factors that are linked to rest intolerance by interacting with specific anxiety symptoms or amplifying perceived time poverty.

Although theoretical connections among these variables exist, traditional methods (e.g., latent variable models or mediation analysis) struggle to reveal their complex interactive conditional associations. These methods assume symptoms are passive reflections of latent constructs, focusing on linear relationships between total scores. While revealing macro-level associations, this approach masks micro-symptom interactions. For instance, does “Academic and Leisure Conflict” directly connect to “Negative Feelings” in rest intolerance, or does it operate via “State Anxiety”? Which personality dimension constitutes the psychological network's core vulnerability? Traditional statistics cannot easily answer these refined mechanistic questions.

Network Analysis offers a novel perspective. It studies relationships and interactions among system elements (Fried et al., 2020). While Mixed Graphical Models (MGM) are advantageous for mixed-type data, Gaussian Graphical Models (GGM) combined with the graphical LASSO (gLASSO) and EBIC provide a highly robust and well-established framework for estimating regularized partial correlation networks, especially when psychological variables are continuously or ordinally scored with sufficient categories (Epskamp et al., 2012). Therefore, this study employed GGM to estimate the primary network topology. The network perspective posits that psychological constructs are dynamic systems of interacting symptoms (nodes) and correlations (edges), rather than passive symptom collections. This allows the identification of high-Centrality core symptoms closely associated with the overall network, and Bridge Symptoms connecting different domains (Cernis et al., 2021). Wang et al. (2025) utilized Exploratory Graph Analysis (EGA) to reveal rest intolerance's internal structure, laying the groundwork for a comprehensive network model incorporating personality, anxiety, and time poverty. Moreover, Yang et al. (2025) validated this method's effectiveness in exploring cross-domain mechanisms within anxiety and personality networks.

In summary, this study constructs a psychological network of personality traits, state anxiety, perceived time poverty, and rest intolerance to identify core and bridge nodes, clarifying their complex relationships. Specifically, we attempt to answer:

*Q1. What are the core components of personality traits, perceived time poverty, and rest intolerance within this network?*
*Q2. Which components exhibit high bridge centrality across these distinct psychological domains?*

This study pioneers the analysis of rest intolerance associations from a symptom network perspective, overcoming the limitations of macro-level traditional research. It theoretically clarifies the dynamic relationships among these variables and provides educators and mental health professionals with precise intervention targets—blocking key nodes or bridge pathways—to effectively improve college students' rest quality and mental health.

## 2 Methods

### 2.1 Participants

Using convenience sampling, we surveyed undergraduates at a university in Anhui Province via the WenJuanXing online platform (www.wjx.com). Of 589 collected questionnaires, 541 valid responses remained after excluding patterned or inaccurate answers (response rate: 91.85%). Participants' mean age was 20.18 years (SD = 1.34, range = 18–25). The sample comprised 153 males (28.3%) and 388 females (71.7%). The Research Ethics Committee of Anqing Normal University approved the study (Ethic Number: No. AQNU2025069), and all participants provided informed consent.

### 2.2 Measures

#### 2.2.1 Demographic variables

Participants reported their demographic variables, including gender, age, major category, only-child status, and place of origin.

#### 2.2.2 Big Five personality traits

The Chinese Big Five Personality Inventory-15 (CBF-PI-15) developed by Zhang et al. (2019) was used to measure individuals' personality traits. The scale includes five dimensions: Neuroticism (N), Conscientiousness (C), Agreeableness (A), Openness (O), and Extraversion (E). Items were rated on a six-point Likert scale ranging from 1 (completely disagree) to 6 (completely agree), with items two and five reverse-scored. Scores for each dimension were calculated by summing the scores of the corresponding items. In this study, the Cronbach's α coefficients for N, C, A, O, and E were 0.865, 0.752, 0.759, 0.864, and 0.703, respectively.

#### 2.2.3 State anxiety

The State Anxiety subscale of the State-Trait Anxiety Inventory (STAI), revised by Li and Qian (1995), was used to assess individuals' state anxiety levels. The scale contains 20 items (10 describing negative emotions and 10 describing positive emotions). Each item is rated on a 4-point Likert scale ranging from 1 (not at all) to 4 (almost always). Positive emotion items are reverse-scored, with higher scores indicating higher levels of state anxiety. Although the scale comprises both direct negative affect items and reverse-scored positive affect items, we utilized the aggregate total score rather than splitting the scale into two distinct nodes. This decision aligns with the established unidimensional theoretical structure of the STAI-State subscale, where reverse-worded items serve primarily to mitigate acquiescence bias rather than representing a distinct psychological construct. The scale was revised for Chinese college student norms by Li and Qian (1995) and has demonstrated good reliability and validity. In this study, the Cronbach's α coefficient for the scale was 0.928.

#### 2.2.4 College students' perceived time poverty

The College Students' Perceived Time Poverty Scale (CSPTP), developed by Cheng et al. (2025), was used to assess individuals' subjective degree of time poverty. The scale includes four dimensions: Time Pressure, Personal Time Needs, Academic and Leisure Conflict, and Time Urgency. Items are rated on a five-point Likert scale ranging from 1 (strongly disagree) to 5 (strongly agree). Scores for each dimension are the sum of the corresponding items, and the total score is the sum of the dimension scores, with higher total scores indicating higher levels of perceived time poverty. In this study, the total Cronbach's α coefficient for the scale was 0.939, and the coefficients for the four dimensions were 0.901, 0.832, 0.892, and 0.813, respectively.

#### 2.2.5 Rest intolerance

The Rest Intolerance Scale-Short Form (RIS-8), developed by Wang et al. (2025), was used to assess individuals' rest intolerance. The scale includes four dimensions: Negative Feelings, Social Comparison, Obsessive Thinking, and Cognitive Bias. Items are rated on a 5-point Likert scale ranging from 1 (strongly disagree) to 5 (strongly agree). In this study, the total Cronbach's α coefficient for the scale was 0.911, and the coefficients for the dimensions were 0.852, 0.871, 0.848, and 0.541, respectively. Given the unacceptably low internal consistency of the Cognitive Bias dimension in this sample (α < 0.60), and following methodological recommendations to avoid unmodeled measurement error in network analysis, this dimension was entirely excluded from all subsequent analyses. Thus, the rest intolerance community in our network comprised three robust nodes (Negative Feelings, Social Comparison, and Obsessive Thinking).

### 2.3 Statistical analysis

Descriptive statistics, Cronbach's α, and correlation analysis among the main variables were conducted using SPSS 27.0. Network analysis was performed using packages such as *networktools, qgraph, bootnet*, and *mgm* in R 4.5.2.

To elucidate the structural relationships among the dimensions, we estimated a regularized partial correlation network. While network analysis is frequently applied at the item level to explore fine-grained symptom interactions, we deliberately constructed our network at the dimension (subscale) level for several theoretical and methodological reasons. First, our primary theoretical objective was to elucidate the overarching interactive pathways and cross-domain bridge connections among distinct psychological constructs (Big Five personality traits, perceived time poverty, state anxiety, and rest intolerance), rather than mapping the internal micro-structure of a single psychometric scale. Dimension-level nodes provide a more parsimonious and theoretically interpretable framework for this purpose. Second, integrating four broad constructs at the item level would result in a highly dense network comprising over 50 nodes. Given our sample size (*N* = 541), a 14-node dimension-level network mathematically ensures substantially higher statistical power, parameter accuracy, and centrality stability (Epskamp et al., 2018). To ensure that these dimension-level nodes represent distinct psychological components rather than mere conceptual overlap, multicollinearity and redundancy were carefully monitored.

This analysis was conducted on 13 nodes. According to previous recommendations (Epskamp et al., 2018), the sample size of 541 is well within the adequate range for a 14-node network estimation. To address potential confounding effects of demographic variables on psychological constructs, the effects of five covariates (gender, age, major, only-child status, and area) were strictly controlled by extracting regression residuals prior to network estimation. The selection of these specific covariates was theoretically driven; for instance, previous research indicates that gender and academic background significantly influence susceptibility to leisure guilt and time pressure under academic expectations (Koo, 2023). However, recognizing that residualization might potentially alter the correlational structure, we also estimated an unadjusted network as a sensitivity analysis (see Section 3.3.8).

The network construction employed the Graphical LASSO algorithm combined with the Extended Bayesian Information Criterion (EBIC), with the tuning parameter (γ) set to the default of 0.5, and correlations were computed using the cor_auto function to handle potentially non-normal data. Notably, the mgm package was exclusively utilized to compute node predictability (*R*^2^), while the primary network structure was estimated via the qgraph package.

## 3 Results

### 3.1 Common method bias test

Using uniform Likert scales in surveys may cause common method bias(CMB, Tehseen et al., 2017). To mitigate this issue from self-reported data, we utilized varied measurement scales (Podsakoff et al., 2012). Harman's single-factor test was also employed to assess CMB. Results indicated 13 factors with eigenvalues >1, with the first factor explaining 29.15% of the variance. Although this value falls below the conventional 40% critical threshold—indicating that a single factor does not account for the majority of the covariance—recent methodological literature highlights that Harman's single-factor test is insufficient to definitively rule out common method bias. Therefore, while CMB may not be the sole driver of the observed variance, its potential influence cannot be entirely excluded given the cross-sectional, self-report nature of our data.

### 3.2 Descriptive statistics and correlations

The means (*M*), standard deviations (SD), and correlation analysis of all variables among the participants in this study are presented in Table 1.

**Table 1:** Descriptive statistics and Pearson correlations for variables.

Variable	*M* ±*SD*	1	2	3	4	5	6	7	8	9	10	11	12
1. N	11.16 ± 3.39	–											
2. C	12.55 ± 2.68	0.05	–										
3. A	13.29 ± 2.56	−0.10^*^	0.37^***^	–									
4. O	11.52 ± 3.02	−0.04	0.44^***^	0.39^***^	–								
5. E	10.29 ± 3.01	−0.38^***^	0.05	0.25^***^	0.21^***^	–							
6. NF	5.70 ± 2.17	0.555^***^	0.014	−0.072	0.012	−0.301^***^	–						
7. SC	6.23 ± 2.09	0.501^***^	0.097^*^	−0.054	0.031	−0.212^***^	0.687^***^	–					
8. OT	6.43 ± 2.02	0.473^***^	0.078	−0.008	0.009	−0.224^***^	0.786^***^	0.692^***^	–				
9. SAI	41.37 ± 9.58	0.623^***^	−0.235^***^	−0.370^***^	−0.174^***^	−0.359^***^	0.543^***^	0.435^***^	0.416^***^	–			
10.TP	24.73 ± 6.51	0.614^***^	−0.076	−0.126^**^	−0.030	−0.353^***^	0.608^***^	0.556^***^	0.549^***^	0.625^***^	–		
11.PT	15.13 ± 4.29	0.440^***^	0.046	−0.043	0.106^*^	−0.247^***^	0.544^***^	0.457^***^	0.475^***^	0.431^***^	0.649^***^	–	
12.AL	12.27 ± 3.61	0.402^***^	0.121^**^	−0.039	0.162^***^	−0.208^***^	0.497^***^	0.483^***^	0.485^***^	0.390^***^	0.608^***^	0.721^***^	–
13.TU	12.48 ± 3.34	0.392^***^	0.042	−0.036	0.099^*^	−0.176^***^	0.435^***^	0.434^***^	0.439^***^	0.371^***^	0.507^***^	0.593^***^	0.549^***^

^*^p < 0.05, ^**^p < 0.01, ^***^p < 0.001; N, Neuroticism; C, Conscientiousness; A, Agreeableness; O, Openness; E, Extraversion; SAI, State Anxiety; NF, Negative Feelings; SC; Social Comparison; OT, Obsessive Thinking; SAI, State Anxiety; TP, Time Pressure; PT, Personal Time Needs; AL, Academic and Leisure Pressure; TU, Time Urgency.

### 3.3 Network analysis among personality traits, anxiety, perceived time poverty, and rest intolerance

To elucidate the structural relationships among the dimensions of personality traits, state anxiety, perceived time poverty, and rest intolerance, we estimated a regularized partial correlation network. This analysis was conducted on 13 nodes. The effects of five covariates—gender, age, major, only-child status, and area—were strictly controlled by extracting regression residuals from multiple linear regression models prior to network estimation.

**Figure 1:**
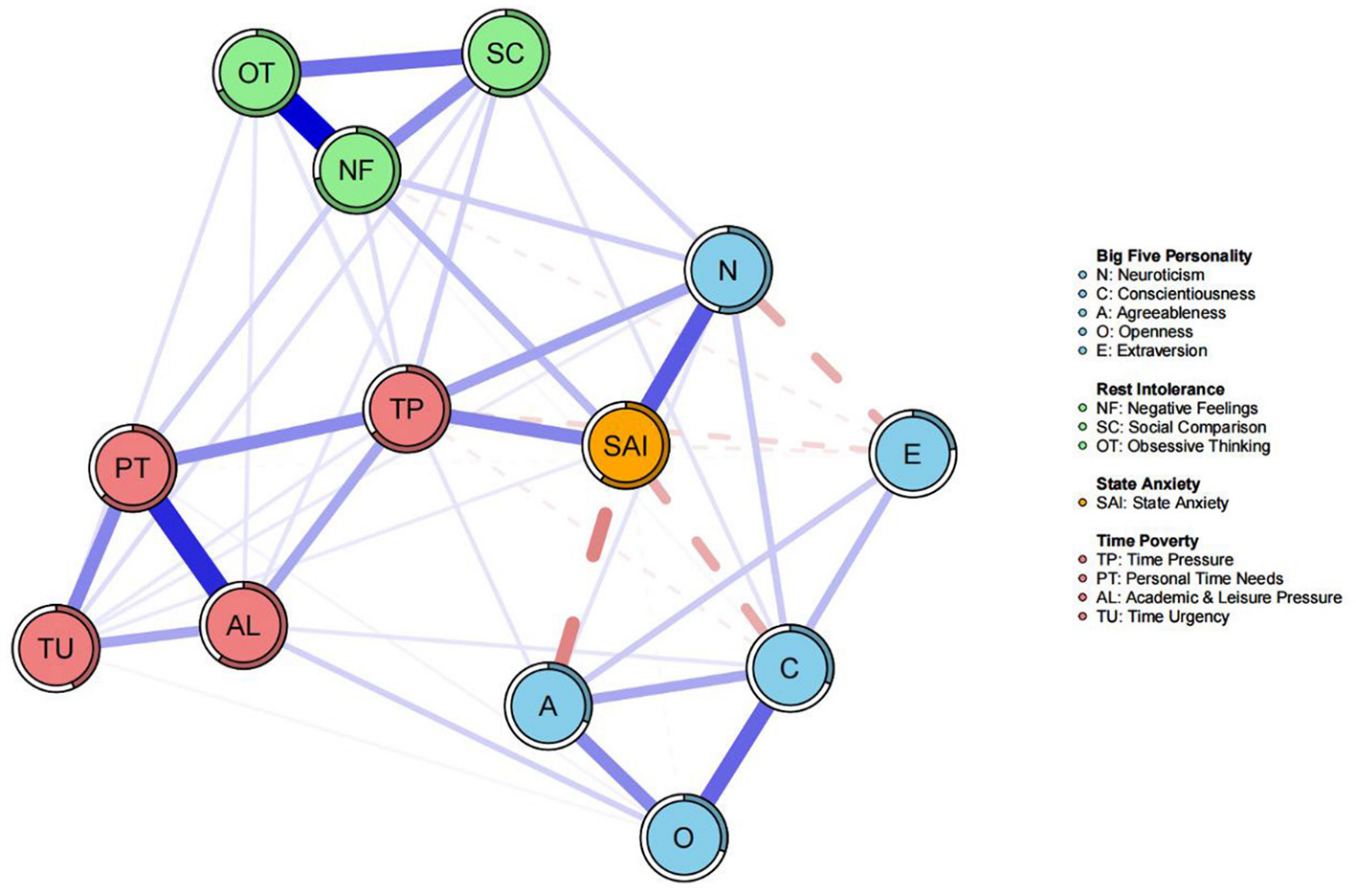
Network Analysis Results. Nodes represent specific psychological dimensions, and edges represent the partial correlations between nodes. Blue edges indicate positive correlations, and red edges indicate negative correlations; the thickness and color saturation of the edges reflect the strength of the associations.

The final network contained 49 non-zero edges out of 78 possible edges, with an average edge weight of 0.058. The network exhibited a moderately high density, reflecting extensive intrinsic connections among the selected psychological constructs. However, as suggested by the output results, the interpretation of weaker edges should remain cautious. Therefore, the discussion in this study will primarily focus on those connections that are significantly strong and theoretically meaningful, as well as the nodes with the highest centrality. The overall structure of the network is shown in Figure 1.

The complete edge-weight matrix and the exact numerical values for all centrality indices (node strength, expected influence, bridge strength) and predictability (*R*^2^) are provided in Supplementary Tables S1 and S2.

After controlling for multiple demographic variables, several key edges still demonstrated particularly strong connections. Notably, there was a strong positive correlation between Neuroticism (*N*) and State Anxiety (SAI) (edge weight = 0.326), representing the strongest connecting channel between the personality traits community and other nodes. Furthermore, in cross-community connections, the link between Time Pressure (TP) and State Anxiety (SAI) was the most significant (edge weight = 0.242), and the connection between State Anxiety (SAI) and Negative Feelings (NF) was also relatively important (edge weight = 0.136). These specific connections clearly outline the main pathways through which the Big Five personality traits and perceived time poverty are associated with the rest intolerance experience via the hub of state anxiety.

#### 3.3.1 Network stability and accuracy

The robustness of the estimated network was rigorously assessed through non-parametric bootstrapping with 1,000 samples. The bootstrapped confidence intervals for the edge weights were generally narrow, confirming the accuracy of the estimated edge parameters (Figure S1).

A case-dropping bootstrap procedure was used to assess the stability of the centrality indices. The results showed that our metrics of interest exhibited excellent stability. The correlation stability coefficients (CS-coefficients) for node strength, expected influence, and bridge strength all reached 0.75, respectively (see Figure S2). These values far exceed the recommended threshold of 0.5, indicating that the ranking of key nodes in the network remained highly stable even after dropping a large portion of the sample, thus confirming the robustness of the centrality estimations.

Additionally, bootstrapped difference tests confirmed that the estimated edge weights and bridge centrality values were statistically distinguishable. The strongest edges in the network were significantly stronger than many weaker edges (Figure S3), and the bridge strength of the most central nodes was significantly higher than that of other nodes in the network (Figure S4). Together, these verify the robustness and interpretability of our findings.

#### 3.3.2 Node predictability

Network predictability varied widely, ranging from 0.232 for Extraversion to 0.717 for Negative Feelings. In network theory, nodes with high predictability (e.g., Negative Feelings, *R*^2^ = 0.717; Obsessive Thinking, *R*^2^ = 0.678) are typically regarded as being highly “determined” by the system, indicating that they function more as highly explained nodes within the network. Conversely, nodes with lower predictability (e.g., Extraversion, *R*^2^ = 0.232; Openness, *R*^2^ = 0.302) are less constrained by neighboring nodes, suggesting they are less explained by neighboring nodes.

#### 3.3.3 Within-community centrality analysis

To address our first research question regarding the core components of personality traits, perceived time poverty, and rest intolerance, we examined the conventional centrality indices—specifically node strength and expected influence—for all nodes within their respective communities. Node strength reflects the sum of the absolute weights of edges connected to a node, while expected influence accounts for the sign of the edge weights, providing a comprehensive measure of a node's relative importance within the estimated network topology.

Within the “Big Five Personality” community, Neuroticism (N) exhibited the highest node strength and expected influence, indicating that it is the most central trait within the personality sub-network. In the “Perceived Time Poverty” community, Time Pressure (TP) emerged as the core node with the highest centrality, suggesting it is the primary cognitive component underlying college students' time poverty experience. Finally, within the “Rest Intolerance” community, Negative Feelings (NF) demonstrated the highest node strength and expected influence. This firmly identifies Negative Feelings as the affective core strongly associated with the rest intolerance experience.. (Note that State Anxiety was evaluated as a single-node community). These centrality results provide a microscopic view of the structural core of each construct, successfully answering our first research question and laying the groundwork for understanding how these communities interact.

#### 3.3.4 Bridge centrality analysis

To address our second research question (Q2) and identify the key nodes connecting different psychological concept communities, we conducted a bridge centrality analysis. Four specific communities were defined for this analysis: “Big Five Personality” (N, C, A, O, E), “State Anxiety” (SAI), “Perceived Time Poverty” (TP, PT, AL, TU), and “Rest Intolerance” (NF, SC, OT). This structural division was strictly theoretically driven: personality traits represent distal stable dispositions; perceived time poverty reflects cognitive appraisals of environmental demands; and rest intolerance constitutes a specific, context-bound symptom cluster. Crucially, State Anxiety was deliberately specified as a distinct, single-node community because it theoretically represents a transient, generalized affective arousal state that mediates the relationship between stable traits, cognitive appraisals, and specific resting distress. As confirmed by our subsequent sensitivity analysis (Section 3.3.5), its prominent bridging role is not a methodological artifact of this community definition.

**Figure 2:**
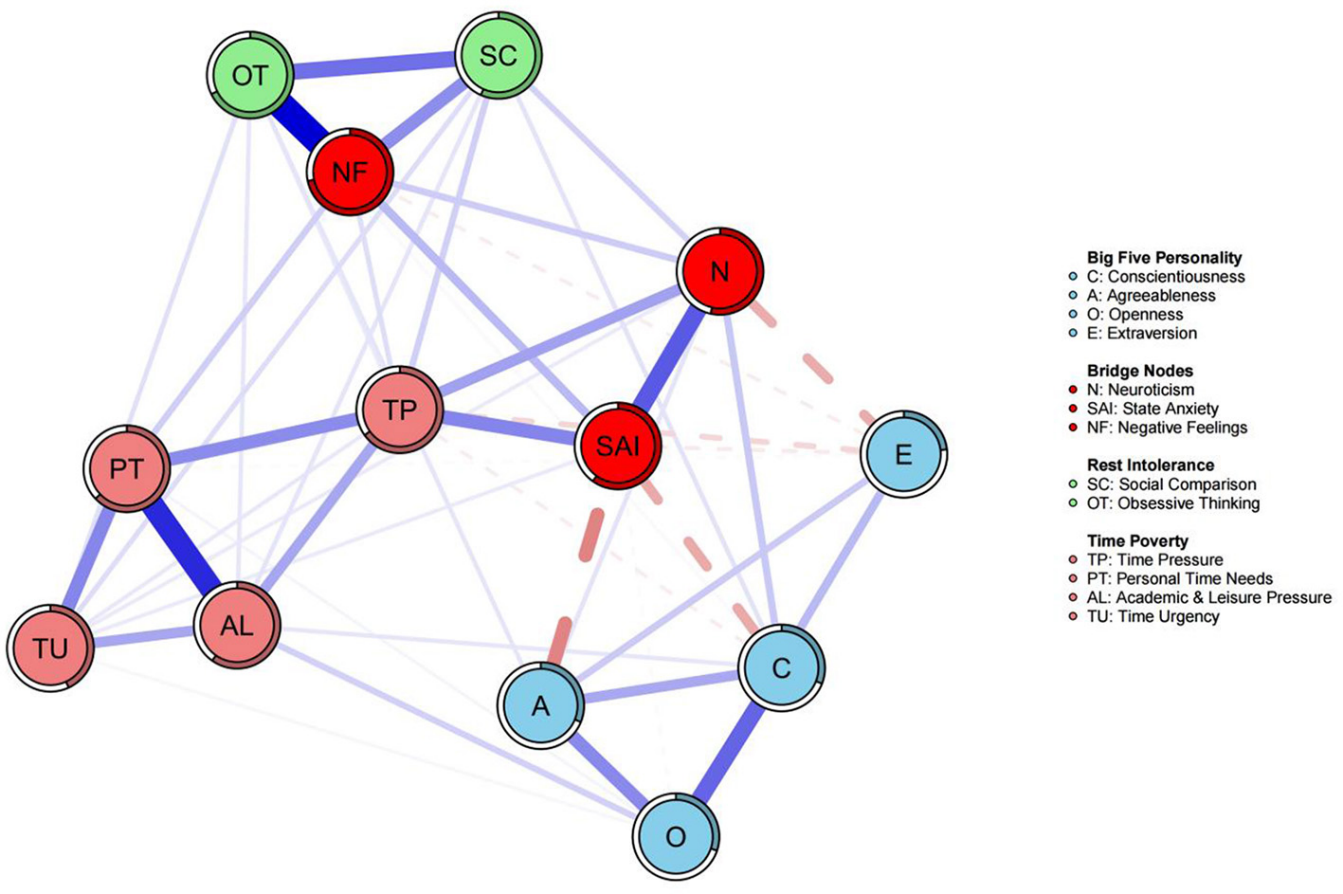
Bridge Network Analysis Results. Nodes marked in red represent the identified core bridge nodes (using the Top 20% threshold of bridge strength), which are Neuroticism (N), State Anxiety (SAI), and Time Pressure (TP). These nodes act as key hubs connecting different psychological communities. Other nodes are colored according to their respective psychological domains. The thickness of the edges represents the strength of the connection.

**Figure 3:**
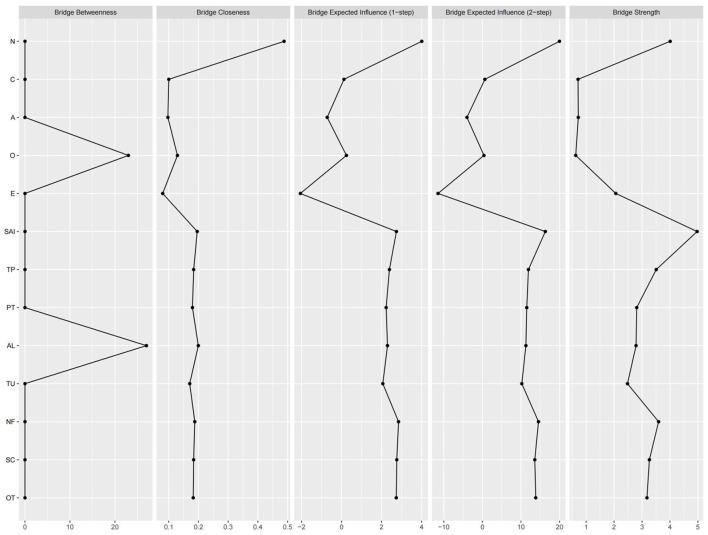
Bridge Centrality Plot. N, Neuroticism; C, Conscientiousness; A, Agreeableness; O, Openness; E, Extraversion; SAI, State Anxiety Inventory; TP, Time pressure; PT, Personal time needs; AL, Academic and Leisure conflict; TU, Time urgency; NF, Negative feelings; SC, Social comparison; OT, Obsessive thinking; CB, Cognitive bias.

The results are visualized in Figure 2, where nodes marked in red were identified as core bridge nodes (using the Top 20% threshold, a conventional criterion in network literature to highlight the most prominent bridging elements; e.g., Jones et al., 2021). The bridge centrality plot (see Figure 3) shows that, based on the specific values of Bridge Strength, the top bridge nodes were: State Anxiety (SAI; Bridge Strength = 4.97), Neuroticism (*N*; Bridge Strength = 4.01), Negative Feelings (NF; Bridge Strength = 3.59), and Time Pressure (TP; Bridge Strength = 3.51).

This analysis strongly confirms that State Anxiety is the most important “transit hub” in the entire psychological network, possessing the highest bridge strength and connecting different communities. Neuroticism serves as the primary gateway from personality traits to the external system, while Time Pressure acts as the core outlet through which perceived time poverty influences other psychological dimensions. These findings directly and successfully answer our second research question, delineating that state anxiety, neuroticism, negative feelings, and time pressure play crucial bridging roles in the pathological interactions among personality, time cognition, and rest intolerance.

#### 3.3.5 Sensitivity analysis for state anxiety

Given that'State Anxiety' (SAI) was classified as a standalone community containing only one node, its bridge strength might be mathematically inflated since all its adjacent edges are inherently cross-community edges. To ensure that the high bridge centrality of SAI was not a methodological artifact, we conducted a sensitivity analysis by temporarily merging SAI into the “Rest Intolerance” community. The re-estimated bridge centrality revealed that although SAI's absolute bridge strength naturally decreased (from 4.97 to 3.57), it firmly remained one of the top three bridge nodes in the entire network (following Neuroticism and Time Pressure). This sensitivity test strongly demonstrates that the critical bridging role of state anxiety is highly robust and reflects a substantive psychological mechanism rather than a statistical artifact.

#### 3.3.6 Supplementary analysis

To enhance the robustness of our findings, we further estimated an alternative network structure in which the four dimensions of rest intolerance (NF, SC, OT, CB) were aggregated into a single total score (RI), and the four dimensions of perceived time poverty (TP, PT, AL, TU) were aggregated into a single total score (CSPTP). The resulting network is shown in Figure S5.

This simplified network contains eight nodes and 26 non-zero edges out of 28 possible edges, with an average edge weight of 0.078. The high density of this network indicates that, at the total-score level, the major psychological constructs are broadly connected. Although the total-score network inevitably obscures fine-grained pathways between specific dimensions (such as “Time Pressure” and “Negative Feelings”), its dense structure macroscopically validates the high degree of association and internal consistency of these constructs as a holistic system. This mutually corroborates the extensive bridging phenomena we observed at the micro (dimension) level, thereby confirming the overall robustness of our primary network findings.

#### 3.3.7 Sensitivity analysis for covariate adjustment

To explicitly address concerns that the residualization of demographic covariates might artificially induce artifacts or remove theoretically relevant variance, we estimated an additional unadjusted network using the raw, non-residualized data. The structural comparison revealed that both the adjusted and unadjusted 13-node networks identified exactly 49 non-zero edges out of 78 possible edges, and their overall topological structures were nearly identical. Crucially, the correlation between the edge-weight matrices of the two networks was remarkably high (Pearson *r* = 0.999, Spearman ρ = 0.998; see Figure S6). This robust consistency confirms that our primary 13-node network structure reflects substantive psychological associations rather than methodological artifacts induced by the covariate control procedure.

## 4 Discussion

This study employed network analysis techniques to construct a comprehensive psychological network comprising personality traits, state anxiety, perceived time poverty, and rest intolerance. By examining the Strength and Expected Influence of the nodes, we identified the core components that exhibit high centrality within each sub-network, and further revealed the interactive pathways among constructs through bridge nodes. This finding not only answers the fundamental question of “what factors constitute the psychological dilemma of rest for college students,” but also provides empirical evidence for understanding its underlying dynamic mechanisms.

### 4.1 Core components of personality traits, perceived time poverty, and rest intolerance

In the personality traits sub-network, Neuroticism was identified as the core element. This finding aligns with broader transdiagnostic perspectives on personality pathology (Widiger and Oltmanns, 2017), indicating that neuroticism serves as a fundamental, trait-like vulnerability factor in individuals' psychopathological networks. As a biological susceptibility to negative stimuli, neuroticism is not directly equivalent to specific psychological symptoms, but rather provides an unstable emotional background for the occurrence of rest intolerance. For college students with high neuroticism, their internal “negative vigilance” mechanism makes them more likely to detect potential threat signals (e.g., worries about the future) when facing unstructured rest time, thereby lowering the psychological threshold for switching from tension to relaxation, and making the individual more easily activated by potential stressors.

In the perceived time poverty sub-network, Time Pressure was identified as the core component, revealing the cognitive root of the time poverty experience. According to (Cheng et al. 2025), time pressure reflects an individual's subjective assessment of the imbalance in the “task-time” resource ratio. Unlike objective busyness, time pressure carries a strong sense of urgency and passivity, serving as a central hub within the entire time cognition system. When individuals are chronically subjected to high-intensity evaluations of time pressure, their psychological resources are over-appropriated; this sense of urgency is closely associated with cognitive rejection of resting behaviors, making it difficult for individuals to stop and rest with peace of mind.

In the rest intolerance sub-network, Negative Feelings were identified as the core feature. This result suggests that rest intolerance essentially manifests primarily as an affective disturbance, rather than mere cognitive biases or behavioral compulsions. Although cognitive biases may provide a prerequisite, what is most strongly linked to this distressing state and is linked to subjective distress to the individual are the negative emotional experiences that directly emerge during rest. As pointed out by (Wang et al., 2025), this “leisure guilt” or “rest anxiety” constitutes the core experience of rest intolerance. Negative feelings, acting as the hub in the network, on the one hand are strongly connected to external time pressure and anxiety, and on the other hand are correlated with obsessive thinking and social comparison through emotional rumination effects. In other words, precisely because they experience intolerable negative emotions during rest, college students are forced to avoid this pain by “pretending to be busy” or “refusing to rest,” which is associated with the complete failure of the restorative function of rest.

### 4.2 Bridge nodes connecting the four constructs

First, State Anxiety serves as the main bridge connecting external pressure with internal rest disorders, and this pattern may be understood through the theory of action economy. This theory posits that an individual's psychological state is associated with shifts in the perception of physical or psychological resources (Bagozzi, 2000). Research by Chen et al. (2023) confirmed that anxiety states were associated with exaggerated judgmental biases in individuals' perceptions of physical environment attributes. Drawing on this theory, in the domain of rest, state anxiety may be associated with how individuals perceive the “cost of resting.” College students reporting high anxiety also tended to report overestimate the potential losses brought by stopping to rest (such as the risk of academic failure) and the “psychological weight” of unfinished tasks. State anxiety is directly connected to the Negative Feelings node in the rest intolerance network—this association may reflect that the distress associated with rest is not necessarily tied to rest itself, but rather co-occurs with anxiety-related catastrophizing evaluations of the consequences of rest. State anxiety shows a strong association with the disrupted transition from “physiological stopping” to “psychological relaxation”.

Second, Time Pressure, as another key bridge, may help illustrate how modern “hustle culture” relates to individuals' leisure experiences. According to Tonietto et al. (2021), when individuals view leisure as an interference with productivity, the intrinsic value of leisure appears to be diminished. The network structure in this study shows that time pressure is directly connected to the Social Comparison and Negative Feelings dimensions of the rest intolerance network, suggesting that high-intensity time pressure forces individuals to engage in social comparison with others and generate negative emotions. As Koo (2023) pointed out in their research, rest may no longer viewed as a necessary means of restoring energy, but may instead be framed as “laziness” with moral flaws; that is, time pressure, through its association with moral anxiety about wasting time, is linked to a reduction in the positive emotional experiences (such as a sense of autonomy and relaxation) otherwise brought about by rest, with these instead being replaced by negative emotional experiences such as guilt and unease. This pattern observed for time pressure appears consistent with reduced Autonomy and Detachment elements in the DRAMMA model proposed by Newman et al. (2014), and may reflect a difficulty disengaging from competitive academic concerns even during leisure time.

Third, Neuroticism, identified here as a bridge node spanning the personality and symptom domains, is consistent with the diathesis-stress model, although the present cross-sectional data cannot establish the directionality implied by this model. In the current network, neuroticism shows a strong positive association with state anxiety and is linked to individuals' tendencies toward social comparison during rest. For individuals reporting high neuroticism, periods of rest lacking clear task instructions may be experienced as more distressing than busy working hours, which co-occurs with greater rumination and social comparison around rest—a pattern that may correspond to a self-reinforcing association between resting and anxiety, though longitudinal data would be needed to confirm any such cycle.

### 4.3 Implications and limitations

This study uses network analysis to examine the associations between personality traits and rest intolerance. Unlike traditional latent variable models, network analysis identifies nodes with high centrality and bridge centrality in the estimated network of personality traits and rest intolerance. The findings describe an associative structure for rest intolerance, highlighting Negative Feelings as the node most strongly connected within the rest intolerance community, Time Pressure within the time poverty community, and Neuroticism within the personality community. The co-occurring pattern among Neuroticism, State Anxiety, and Time Pressure is consistent with—though does not by itself demonstrate—a possible route by which negative emotions may be related to rest-related distress through altered resource perception, a hypothesis that would require longitudinal or experimental designs to test directly.

The network structure suggests that Time Pressure and State Anxiety occupy bridge positions connecting distal personality traits and rest intolerance in the estimated network. While these results cannot establish causal intervention targets, they may generate hypotheses for future intervention research. For instance, future intervention studies could explore whether cognitive restructuring approaches addressing the belief that “unstructured time is wasted time” (Tonietto et al., 2021) are associated with reductions in the link between perceived time scarcity and negative feelings during rest. Similarly, given that State Anxiety occupies a central bridging position in relation to the rest intolerance community, future studies might examine whether mindfulness practices (e.g., Acceptance and Commitment Therapy) are associated with reduced co-occurrence of anxiety arousal and compulsive social comparison—though such applications remain speculative pending experimental validation.

Although this study describes an estimated psychological network of rest intolerance among college students, it also has certain methodological limitations. First, its cross-sectional design does not permit causal inference, and the use of a single cohort of Chinese college students limits the generalizability of the findings. Second, the reliance on self-report questionnaires with uniform Likert-type response formats introduces the ongoing risk of common method bias. Although our preliminary test did not indicate that a single factor accounted for the majority of variance, shared method variance might still be associated with inflated observed network edge weights. Third, regarding measurement validity, the Cognitive Bias dimension of the short-form Rest Intolerance Scale exhibited poor internal reliability in our sample (α < 0.60) and was therefore excluded from the network structure. While this conservative decision was intended to reduce unmodeled measurement error in our 13-node model, it also means our network did not fully capture the cognitive bias facet of rest intolerance. Future research should employ longitudinal, multi-informant, or experimental designs to better assess causality, examine the role of method bias, re-evaluate psychometric properties, and explore how factors such as gender, academic stage, and culture relate to the estimated rest intolerance network.

For example, our sample exhibited a significant gender imbalance, with females comprising 71.7% of the participants. This overrepresentation may have shaped the estimated network structure, as research indicates that females, under the dual expectations of family and academics, often report higher levels of leisure guilt (Koo, 2023). It is possible that this demographic characteristic contributed to the edge weights between time pressure and negative feelings in our network. Although conducting a Network Comparison Test by gender would be ideal to explore these topological differences, the highly unequal sample sizes between male (*n* = 153) and female (*n* = 388) groups in our current data would likely result in low statistical power and unreliable comparison metrics. Therefore, future studies with larger, gender-balanced samples are needed to formally test for network invariance. Furthermore, while we controlled for basic demographic variables, our study did not comprehensively capture detailed academic and socioeconomic characteristics, such as specific academic workload, grade point average, or family socioeconomic status. These unmeasured contextual factors are may be related to students' perceived time pressure and rest intolerance. Future research should integrate these variables to provide a more nuanced understanding of the institutional context shaping these psychological networks.

Moreover, cultural backgrounds may be related to the symptom expression of rest intolerance; for instance, Asian students influenced by the Confucian value that “diligence has merit, play has no benefit” might report more cognitive biases and moral anxiety (Huang and Gove, 2015). Therefore, future studies could construct subgroup networks or conduct network comparison tests to examine these differences in network topology and node centrality.

Additionally, regarding measurement validity, the Cognitive Bias dimension of the short-form Rest Intolerance Scale exhibited poor internal reliability in our sample and was therefore excluded from the network structure to reduce the risk of unmodeled measurement error. While this was intended to support the robustness of our 13-node model, it also means our network did not fully capture the cognitive bias facet of rest intolerance. Future studies should re-evaluate the psychometric properties of this specific subscale or utilize alternative instruments with stronger reliability.

## 5 Conclusion

In summary, network analysis has revealed the multidimensional, intertwined relationships among personality traits, state anxiety, perceived time poverty, and rest intolerance, and identified several key nodes, expanding our understanding of the micro-interactive mechanisms among these traits. The study found that the core component of rest intolerance is Negative Feelings, the core of perceived time poverty is the perception of Time Pressure, and Neuroticism is the core of personality traits. The combination of these three forms the core experience of individuals with high neuroticism when coping with resting situations—that is, under the combined context of trait susceptibility and cognitive urgency, rest is no longer a restorative process, but is experienced as an emotional burden filled with guilt and anxiety.

Furthermore, the identification of key bridge nodes, such as Neuroticism, State Anxiety, and Time Pressure, highlights the critical intermediary connections linking broad personality traits to specific rest-related distress. Rather than proposing a direct causal translation, these findings suggest that state anxiety and time pressure serve as key correlates that connect external environmental demands with internal rest intolerance experiences. These results help clarify the ‘trait-cognition-emotion' network interactions linking trait susceptibility, environmental cognitive appraisal, and rest intolerance dimensions, and provide valuable insights for future theoretical construction and precise interventions regarding rest intolerance among college students.

## Data Availability

The raw data and the R scripts used for network estimation and centrality analyses supporting the conclusions of this article are available in the Science Data Bank repository, at doi: 10.57760/sciencedb.39865.
