# Supplementary material for: Bridging personality, time poverty, and rest intolerance: a network analysis
Source: Front Psychol. 2026 Jul 17;17:1872834. doi: 10.3389/fpsyg.2026.1872834 (PMC13424486; doi:10.3389/fpsyg.2026.1872834)
Supplement: Supplementary file 1 [file Supplementary_file_1.docx]

**Table S1: Centrality Indices and Predictability for the 13 Network Nodes**

| **Domain** | **Node** | **Node Strength (Z-score)** | **Expected Influence  (Z-score)** | **Predictability (*R*2)** | **Bridge Strength** |
| --- | --- | --- | --- | --- | --- |
| **Big Five Personality** | Neuroticism | 0.58 | 0.11 | 0.535 | 4.01 |
|  | Conscientiousness | -0.11 | -0.64 | 0.309 | 0.71 |
|  | Agreeableness | -0.51 | -0.94 | 0.312 | 0.72 |
|  | Openness | -0.74 | 0.24 | 0.302 | 0.63 |
|  | Extraversion | -1.90 | -2.29 | 0.232 | 2.06 |
| **State Anxiety** | State Anxiety | 1.38 | -1.18 | 0.599 | 4.97 |
| **Time Poverty** | Time Pressure | 1.22 | 0.70 | 0.651 | 3.51 |
|  | Personal Time Needs | 0.34 | 0.83 | 0.629 | 2.81 |
|  | Academic & Leisure Conflict | 0.30 | 0.86 | 0.598 | 2.79 |
|  | Time Urgency | -1.45 | -0.07 | 0.431 | 2.48 |
| **Rest Intolerance** | Negative Feelings | 1.11 | 1.09 | 0.717 | 3.59 |
|  | Social Comparison | -0.48 | 0.45 | 0.567 | 3.26 |
|  | Obsessive Thinking | 0.26 | 0.84 | 0.678 | 3.18 |

**Table S2: Regularized Partial Correlation Weight Matrix for the Adjusted Network**

|  | **N** | **C** | **A** | **O** | **E** | **SAI** | **TP** | **PT** | **AL** | **TU** | **NF** | **SC** | **OT** |
| --- | --- | --- | --- | --- | --- | --- | --- | --- | --- | --- | --- | --- | --- |
| **N** | - | 0.108 | 0.054 | - | -0.160 | 0.326 | 0.184 | - | - | 0.039 | 0.098 | 0.087 | - |
| **C** | 0.108 | - | 0.170 | 0.305 | -0.024 | -0.167 | -0.036 | - | 0.048 | - | - | 0.055 | 0.012 |
| **A** | 0.054 | 0.170 | - | 0.231 | 0.103 | -0.243 | - | - | - | - | - | - | 0.050 |
| **O** | - | 0.305 | 0.231 | - | 0.135 | -0.011 | - | 0.019 | 0.090 | 0.016 | - | - | - |
| **E** | -0.160 | -0.024 | 0.103 | 0.135 | - | -0.042 | -0.079 | -0.010 | - | - | -0.036 | - | - |
| **SAI** | 0.326 | -0.167 | -0.243 | -0.011 | -0.042 | - | 0.242 | - | - | 0.040 | 0.136 | - | - |
| **TP** | 0.184 | -0.036 | - | - | -0.079 | 0.242 | - | 0.228 | 0.172 | 0.043 | 0.064 | 0.092 | 0.039 |
| **PT** | - | - | - | 0.019 | -0.010 | - | 0.228 | - | 0.422 | 0.240 | 0.094 | - | - |
| **AL** | - | 0.048 | - | 0.090 | - | - | 0.172 | 0.422 | - | 0.172 | - | 0.052 | 0.048 |
| **TU** | 0.039 | - | - | 0.016 | - | 0.040 | 0.043 | 0.240 | 0.172 | - | - | 0.065 | 0.059 |
| **NF** | 0.098 | - | - | - | -0.036 | 0.136 | 0.064 | 0.094 | - | - | - | 0.223 | 0.505 |
| **SC** | 0.087 | 0.055 | - | - | - | - | 0.092 | - | 0.052 | 0.065 | 0.223 | - | 0.282 |
| **OT** | - | 0.012 | 0.050 | - | - | - | 0.039 | - | 0.048 | 0.059 | 0.505 | 0.282 | - |

**Note:** '-' indicates edges that were regularized to zero by the LASSO penalty, implying no direct partial correlation between the two nodes; N, Neuroticism, C, Conscientiousness, A, Agreeableness, O, Openness, E, Extraversion, SAI, State Anxiety, NF, Negative Feelings, SC, Social Comparison, OT, Obsessive Thinking, SAI, State Anxiety, TP, Time Pressure, PT, Personal Time Needs, AL, Academic & Leisure Pressure, TU, Time Urgency.

**Figure S1: Bootstrapped confidence intervals of the estimated edge weights for the primary network.**

**
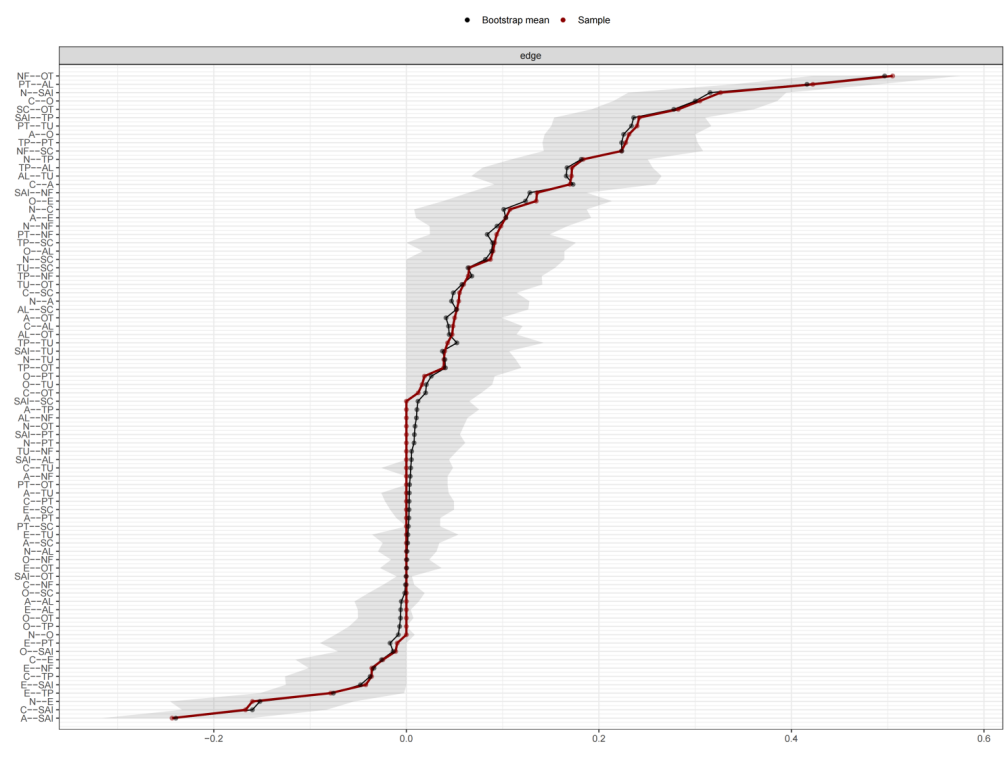
**

**Note:** The red line indicates the sample estimated values, and the gray area indicates the 95% bootstrapped confidence intervals. The narrow intervals confirm the accuracy of the estimated edge parameters.

**Figure S2:** **Centrality stability tested via the case-dropping bootstrap procedure.**

**
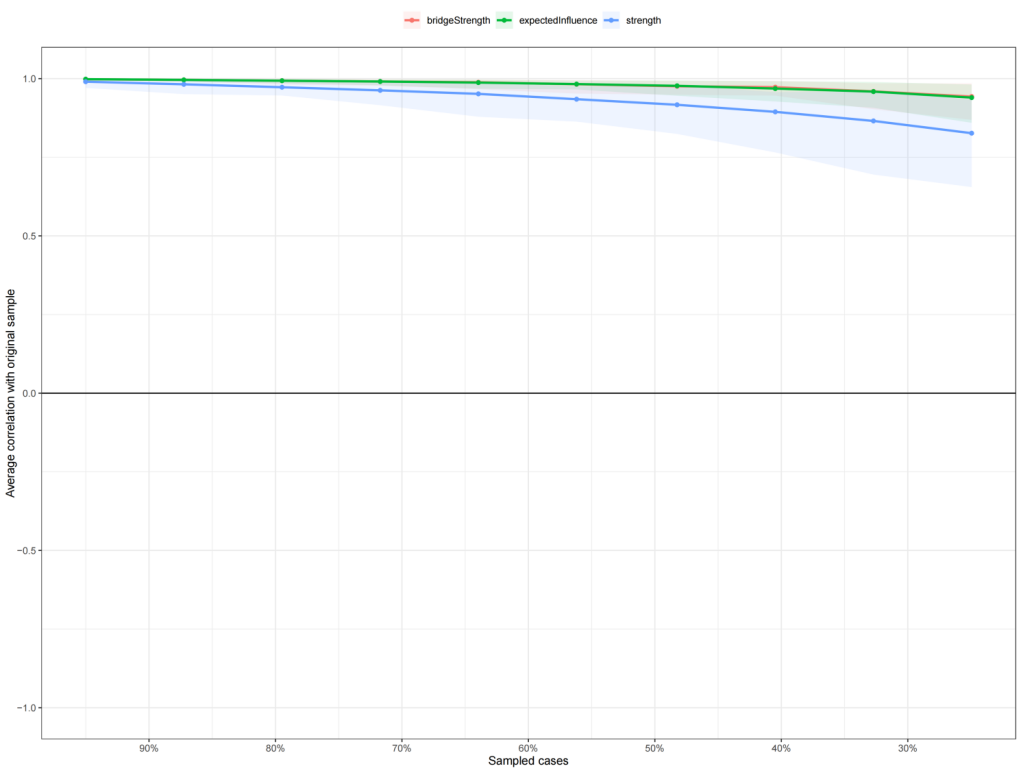
**

**Note:** The lines illustrate the average correlations between centrality indices of sampled networks and the original network as decreasing percentages of cases are sampled. The plot confirms the excellent stability of node strength, expected influence, and bridge strength (CS-coefficients ≥ 0.673).

**Figure S3: Bootstrapped difference tests for edge weights.**

**
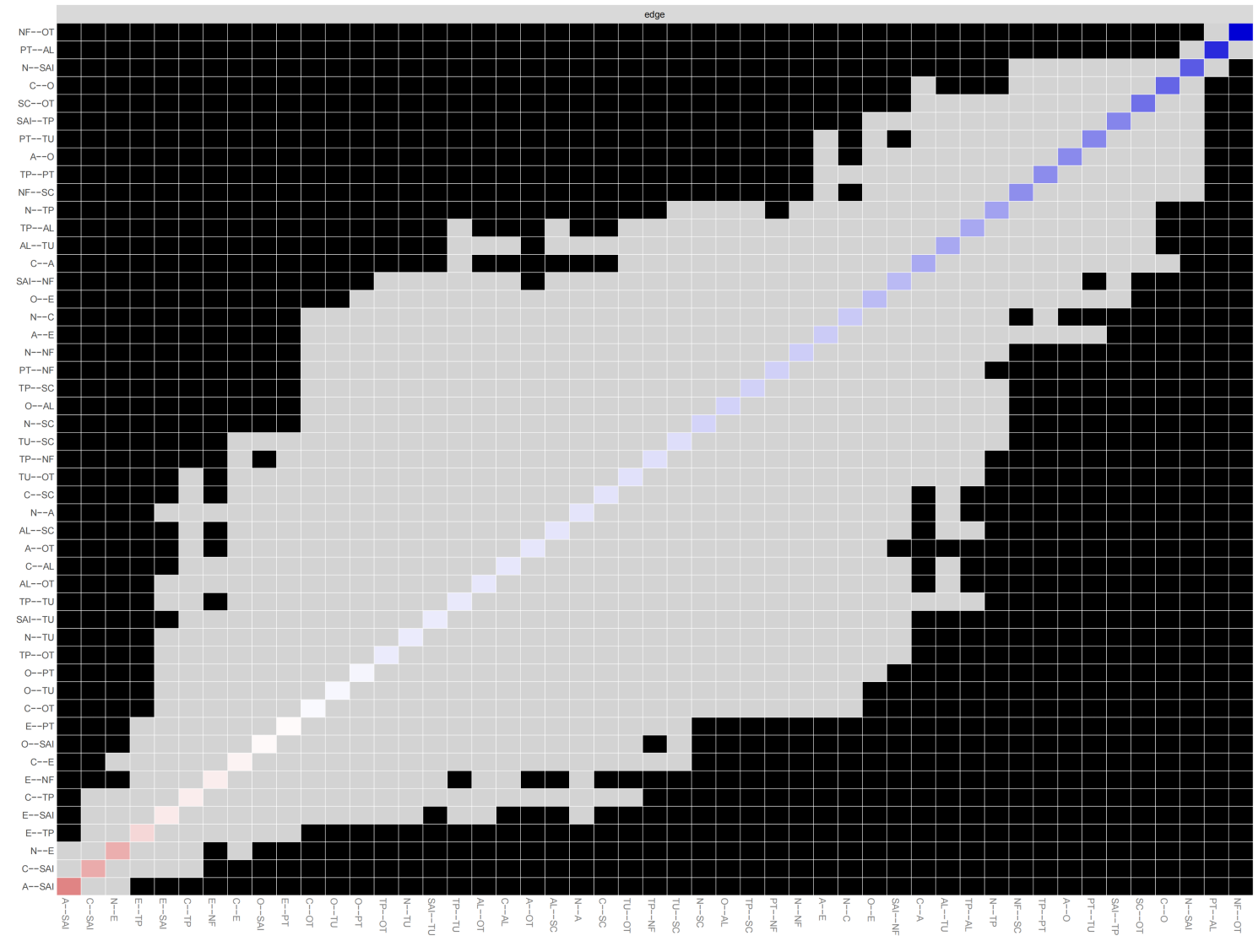
**

**Note:** Black boxes indicate that the two corresponding edge weights differ significantly from one another (α = 0.05), whereas gray boxes indicate that the difference is not statistically significant.

**Figure S4: Bootstrapped difference tests for node bridge strength.**

**
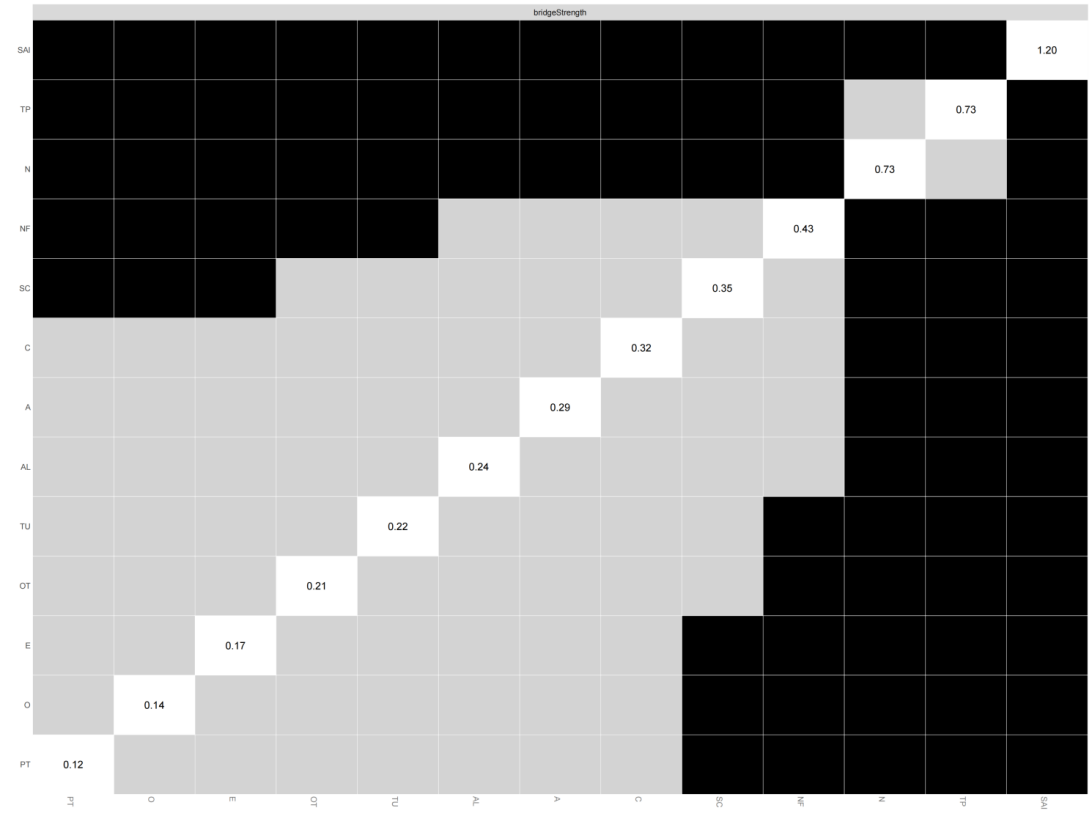
**

**Note:** Black boxes indicate that the bridge centralities of the two corresponding nodes differ significantly from one another (α = 0.05), whereas gray boxes indicate that the difference is not statistically significant.

**Figure S5: Regularized partial correlation network of the aggregated total scores.**

**
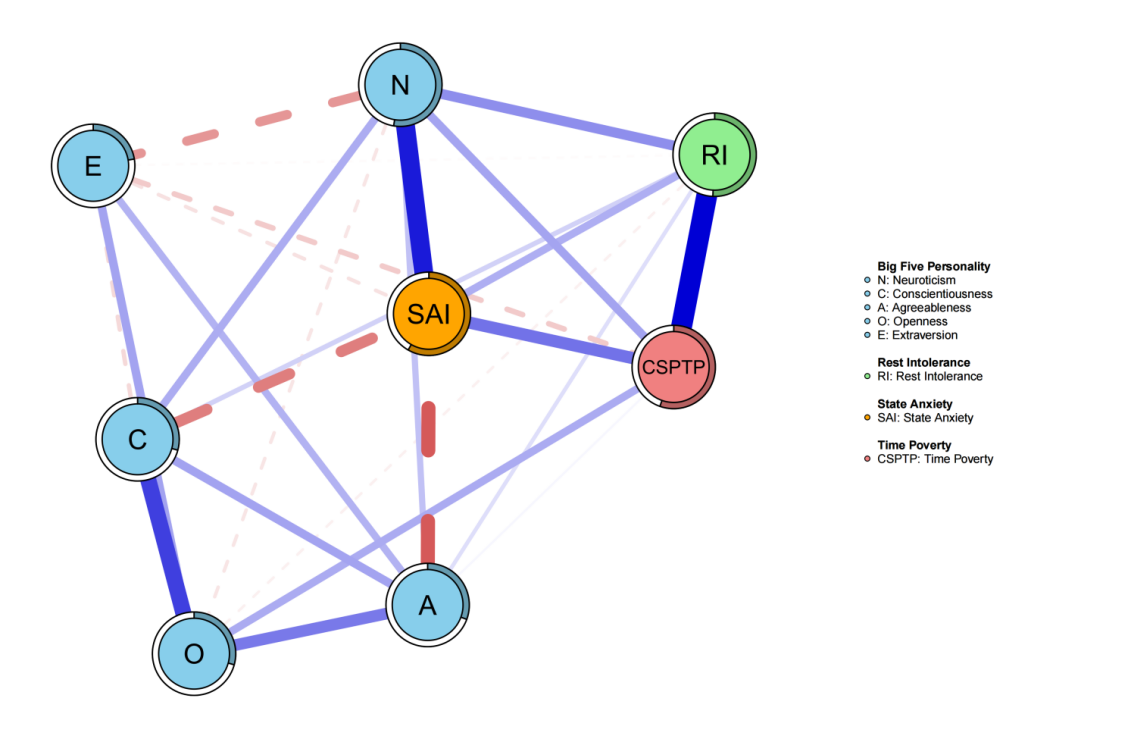
**

**Note:** The four dimensions of rest intolerance were aggregated into a single total score (RI), and the four dimensions of perceived time poverty were aggregated into a single total score (CSPTP). Blue edges indicate positive partial correlations, red edges indicate negative partial correlations, and the thickness of the edges reflects the connection strength.

**Figure S6: Scatter plot comparing edge weights of the covariate-adjusted and unadjusted 13-node networks.**


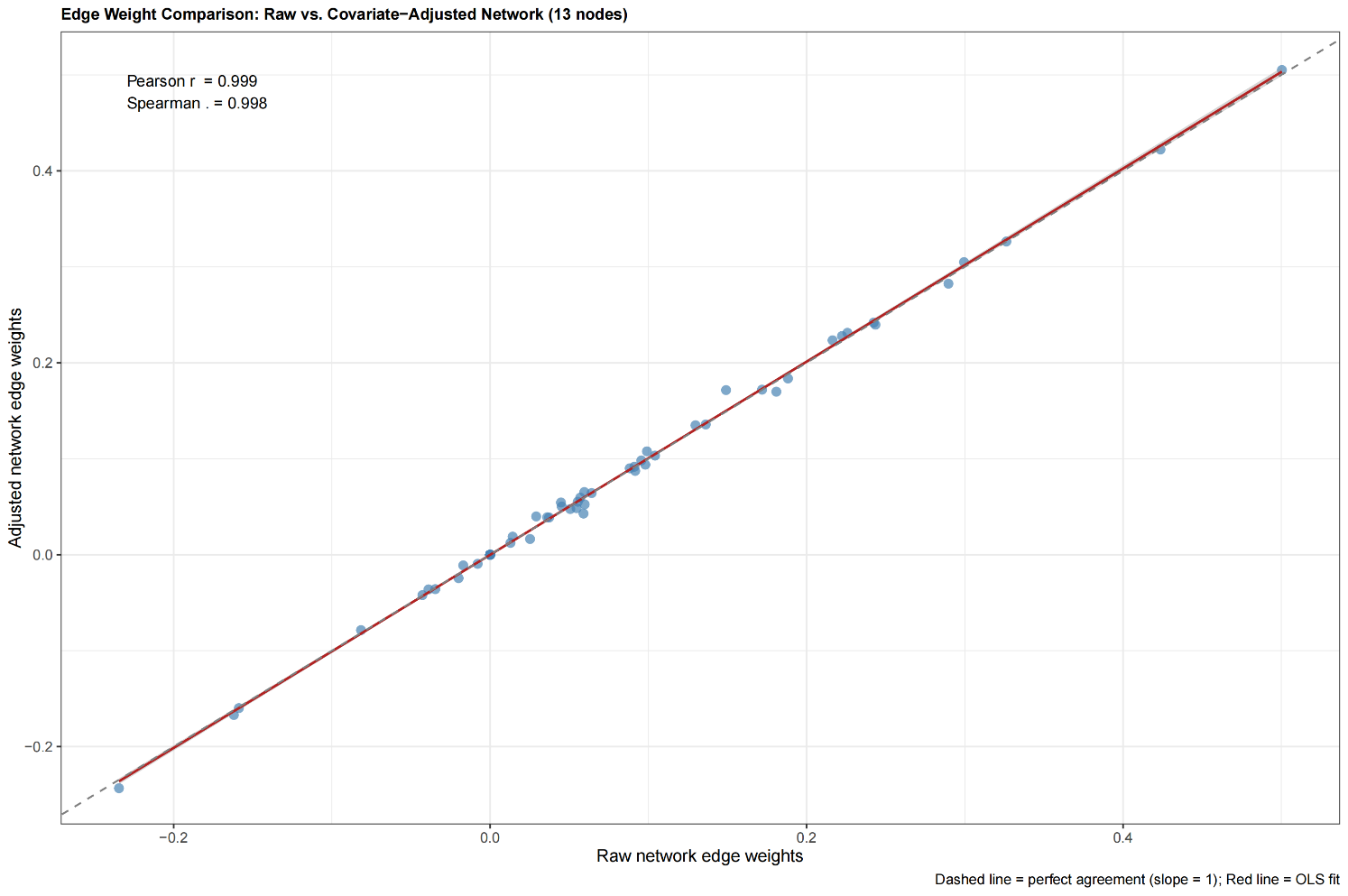


**Note:** Each point represents the weight of a specific edge in the network. The x-axis represents the edge weights from the raw network (without covariate control), and the y-axis represents the edge weights from the adjusted network (with demographic covariates controlled). The dashed line represents perfect agreement (slope = 1), and the solid red line represents the ordinary least squares (OLS) fit. The extremely high correlations (Pearson r = 0.999, Spearman ρ = 0.998) structurally confirm that the residualization of demographic variables did not artificially distort the primary network associations.
